# Quality of Life and Sexual Function after Laparoscopic Posterior Vaginal Plication Plus Sacral Colpopexy for Severe Posterior Vaginal Prolapse

**DOI:** 10.3390/jcm13020616

**Published:** 2024-01-22

**Authors:** Andrea Morciano, Michele Carlo Schiavi, Matteo Frigerio, Giulio Licchetta, Andrea Tinelli, Mauro Cervigni, Giuseppe Marzo, Giovanni Scambia

**Affiliations:** 1 Panico Pelvic Floor Center, Department of Gynaecology and Obstetrics, Pia Fondazione “Card. G. Panico”, 73039 Tricase, Italy; giulio.licchetta@gmail.com (G.L.); g.marzo@piafondazionepanico.it (G.M.); 2 AIUG Research Group, Associazione Italiana di UroGinecologia e del Pavimento Pelvico, 00168 Roma, Italy; michelecarlo.schiavi@gmail.com (M.C.S.); frigerio86@gmail.com (M.F.); mauro.cervigni@libero.it (M.C.); 3 Department of Gynaecology and Obstetrics, “Sandro Pertini” Hospital, 00157 Roma, Italy; 4 Department of Obstetrics and Gynecology, ASST Monza, San Gerardo Hospital, 20900 Monza, Italy; 5 Department of Gynaecology and Obstetrics, “Veris Delli Ponti Hospital”, 73020 Scorrano, Italy; andreatinelli@gmail.com; 6 Department of Urology, Università “La Sapienza”, ICOT-Latina, 00161 Roma, Italy; 7 Department of Gynaecology and Obstetrics, Fondazione Policlinico Universitario “A. Gemelli”, Università Cattolica del Sacro Cuore, 00168 Roma, Italy; giovanni.scambia@policlinicogemelli.it

**Keywords:** posterior vaginal prolapse, posterior plication, sacral colpopexy, laparoscopy, pelvic organ prolapse, quality of life, sexual function, PGI-I, FSDS, PISQ-12

## Abstract

Background: Laparoscopic sacral colpopexy (LSC) is the gold standard treatment for women with apical/anterior pelvic organ prolapse (POP). For isolated posterior vaginal prolapse, instead, the literature suggests fascial native tissue repair. This is a retrospective 2-year quality-of-life follow-up study after laparoscopic posterior plication (LPP) combined with LSC in patients with anterior/apical prolapse combined with severe posterior colpocele. The primary endpoint was to evaluate the subjective outcomes quality of life (QoL), sexual function, and patient satisfaction rate. The secondary endpoint was to evaluate perioperative and anatomical outcomes at the 2-year follow-up. Methods: A total of 139 consecutive patients with anterior and/or apical prolapse (POP-Q stage ≥ II) and severe posterior vaginal prolapse (posterior POP-Q stage ≥ III) were retrospectively selected from our database among women who underwent, from November 2018 to February 2021, a “two-meshes” LSC. The patients were classified into Group A (81 patients; LSC plus LPP) and Group B (67 patients; LSC alone). The primary endpoint was evaluated using the Patient Global Impression of Improvement (PGI-I), the Pelvic Organ Prolapse Distress Inventory-6 (POPDI-6), the Pelvic Floor Impact Questionnaire-7 (PFIQ-7), the Female Sexual Distress Scale (FSDS), the Pelvic Organ Prolapse/Urinary Incontinence Sexual Questionnaire (PISQ-12), and the EuroQol (EQ-5D). The secondary endpoint was studied using the POP-Q study and an intra-, peri-, and post-operative complications assessment. Two-year follow-up data were analyzed for the study. Results: At 2 years, all women showed a statistically significant amelioration of their symptoms on the QoL questionnaires. We found a statistical difference in favor of posterior plication in terms of the PGI-I successful outcome rate (Group A versus B: 85.3% versus 67.1%), FSDS (median 11 versus 21), and PISQ-12 (median 89 versus 62) (*p* < 0.05 for all comparisons). A significant improvement of all EQ-5D values was observed from baseline to 2-year follow-up, and only for the “pain/discomfort” domains did we observe a significant improvement in LSC plus LPP patients versus LSC alone (*p* < 0.05). LSC plus LPP women showed, at 2 years, a significant amelioration of their Ap and GH POP-Q points. We observed no statistical differences in terms of intra-post-operative complications or anatomic failure rate between groups. Conclusions: Our LPP approach to LSC appears to be a safe, feasible, and effective treatment for advanced pelvic organ prolapse with a significant impact on the patient’s general health and sexual quality of life. Adding laparoscopic posterior vaginal plication to “two-meshes” sacral colpopexy is recommended in patients with apical/anterior prolapse and concomitant severe posterior colpocele. This surgical approach, in addition to improving the anatomical results of these patients, is associated with a significant improvement in sexual and quality of life indexes.

## 1. Introduction

Laparoscopic sacral colpopexy (*LSC*) is an effective treatment for women with apical pelvic organ prolapse (*POP*) and was linked to a low risk of anatomic recurrent prolapse if compared with vaginal approaches [1]. However, for posterior vaginal prolapse, data from the literature suggest fascial native tissue repair is superior in terms of recurrence risk [2]. Posterior vaginal plication, routinely vaginally performed, represents, therefore, the most appropriate technique for the treatment of isolated posterior prolapse [2]. What is the best therapeutic option, however, for patients with the simultaneous presence of anterior–apical prolapse, for which the literature recommends an abdominal approach (*LSC*), or severe posterior prolapse, for which the guidelines recommend fascial vaginal surgery?

In 2023, our Center validated *laparoscopic posterior vaginal plication* (*LPP*) combined with standard *LSC* (*LSC plus LPP*) for patients with anterior/apical prolapse associated with severe posterior colpocele, demonstrating significant results in terms of anatomical repair in both the anterior/apical and posterior compartment [3].

In POP surgery, anyway, it is not only important to consider the anatomical repair but also the amelioration of impaired quality of life (*QoL*) and the resolution of sexual dysfunction, both not always reflecting anatomy [1]. Women with pelvic floor dysfunction, such as pelvic organ prolapse or urinary and colorectal–anal symptoms, have a worse perceived quality of life in all dimensions. In particular, prolapse symptoms were found to have the biggest impact on the quality of life and, specifically, on its emotional component [4].

Recent studies have identified that the presence of these pelvic floor disorders and the impact of their symptoms reduce health-related *QoL* in women; likewise, they have direct negative consequences on physical, psychological, sexual, and social health [4]. These disabling problems lead to social isolation, affect the performance of tasks, cause a loss of personal and intimate relationships, and reduce participation in leisure activities [4]. The World Health Organization (WHO) defines “Quality of Life as an individual’s perception of their position in life in the context of the culture and value systems in which they live and in relation to their goals, expectations, standards and concerns” [5]. Consequently, surgeons have increasingly focused their attention on subjective outcomes and *QoL* indicators, rather than simply on vaginal anatomical restoration [6].

In light of these observations, after our previous study on the 1-year follow-up anatomical results after the LPP technique, the aim of the present study was to complete our evaluation of LPP, *primarily* analyzing *QoL*, sexual function, and satisfaction rates among patients who underwent *LPP plus LSC* in our Pelvic Floor Center.

*Secondarily*, we investigated anatomical and perioperative outcomes, with a 2-year follow-up, linked to our surgical technique.

## 2. Materials and Methods

### 2.1. Patient Selection

This is a single-center study conducted at the Pelvic Center of Pia Fondazione “Cardinale G. Panico” of Tricase (Lecce), Italy.

**Figure 1:**
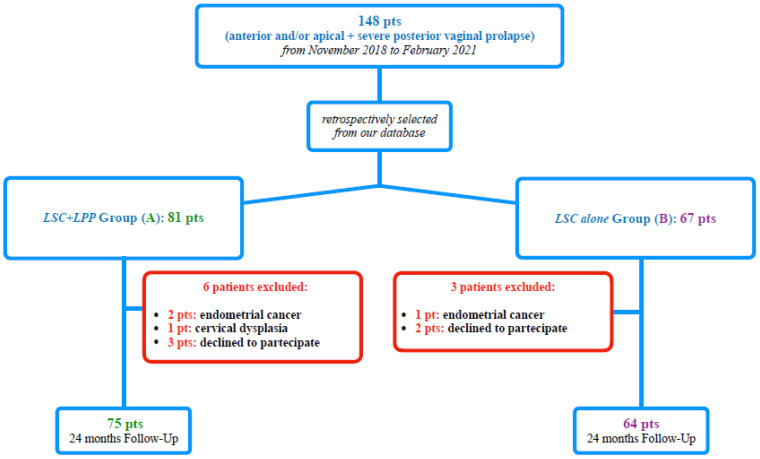
Study flow-chart according to STROBE.

We retrospectively selected from our database 148 consecutive patients with anterior and/or apical POP (POP-Q stage ≥ II) and severe posterior vaginal prolapse (posterior POP-Q stage ≥ III) [7] among women who underwent, from November 2018 to February 2021, a “two-meshes” [3] LSC (Figure 1). In particular, we performed our selection among patients randomly recruited in our previous study [3], with a further extension of selection to February 2021.

We, therefore, selected, according to the surgical approach to posterior vaginal prolapse, 81 patients for Group A (LSC *plus* LPP) and 67 patients for Group B (LSC *alone*). Of the 148 included subjects, 9 were excluded, not meeting the inclusion criteria. All of the remaining 139 patients included in the retrospective study completed our 24-month follow-up (Figure 1).

### 2.2. Surgical Technique

All patients, after laparoscopic supracervical hysterectomy (LSH) and vesico-vaginal preparation, underwent a posterior recto-vaginal dissection.

**Figure 2:**
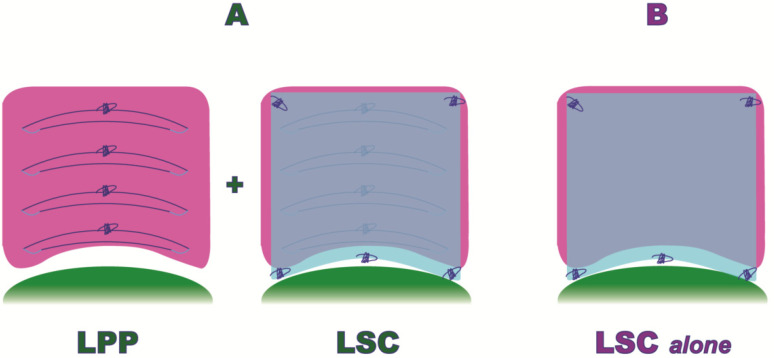
Laparoscopic Posterior Vaginal Fascial Plication and Mesh Fixation.

Only Group A patients underwent, before sacral colpopexy, a laparoscopic posterior plication, as shown in Figure 2.

**Figure 3:**
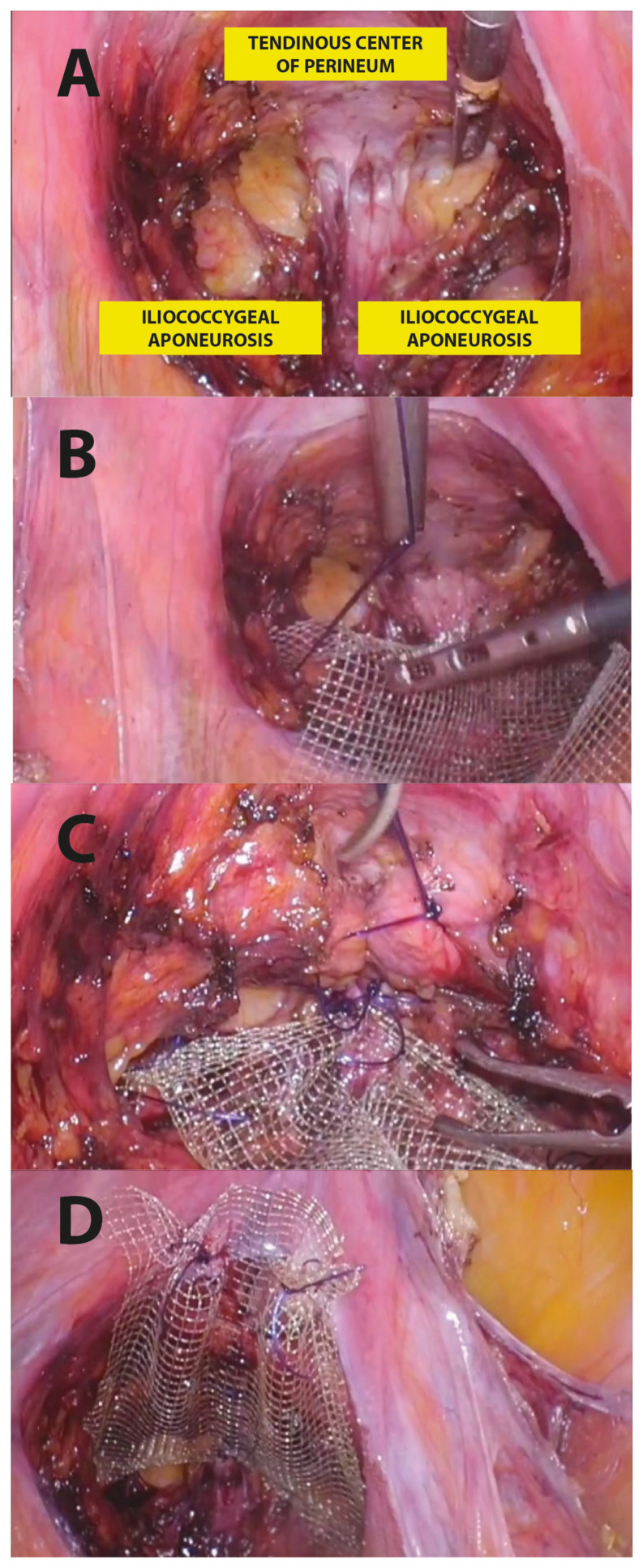
Step-by-step LPP procedure.

In particular, the surgical LPP procedure consisted of the following steps (Figure 3):

*1.*  *Posterior recto-vaginal dissection*: after Douglas peritoneum opening, dissection is carried out lateral to the rectum upward to identify the tendinous center of the perineum and the pelvic parietal fascia covering the levator ani muscle. This allows us to expose the entire vaginal length, with the iliococcygeus muscles laterally and the tendinous center of the perineum medially as the caudal apex, and with the uterine taurus as the cranial apex (Figure 3A).
*2.*  *Posterior mesh fixation* (*caudal sutures*): we fixed mesh caudally to the iliococcygeus muscle aponeurosis and the caudal part of the vagina, near the tendinous center of the perineum (Figure 3B).
*3.*  *Posterior vaginal plication*: this is practiced using 4 extracorporeal interrupted 0 delayed absorbable polydioxanone monofilament sutures (PDO 0, RESORBA Medical GmbH, Nürnberg, Deutschland), placed horizontally, in small steps and in a semicircle and horizontal manner, duplicating the entire vaginal length (Figure 2 and Figure 3C).
*4.*  *Posterior mesh fixation* (*cranial sutures*)*:* the mesh is cranially tension-free fixed to the uterine insertions of the uterosacral ligaments (Figure 3D).

Both populations (Group A and B) underwent an anterior and posterior mesh fixation (Restorelle XL, Coloplast Corp., Minneapolis, MN, USA), (Figure 2).

Anterior meshes alone were, then, fixed to the longitudinal vertebral ligament of the promontory with 2 non-absorbable 0 stitches, and then reperitonealization with 3–0 absorbable barbed sutures was practiced (V-Lock 3.0, Covidien, Dublin, Ireland).

Specimen extractions were finally performed with an extracorporeal transumbilical in-bag manual morcellation. No additional urinary incontinence or POP surgical procedures were practiced.

### 2.3. Data Evaluation

All studied patients underwent a 24-month urogynecological follow-up.

The inclusion criteria for the study were:

- no previous procedures for pelvic prolapse;
- iatrogenic or physiologic menopause;
- age ≤ 80 years;
- POP-Q stage >II for the anterior and/or apical compartment and ≥III for the posterior compartment [7];
- no defecatory dysfunction;
- no uterine size larger than conforming to 10 weeks gestation;
- no endometrial disorders or uterine cervical dysplasia.

Defecatory dysfunction was assessed by patient-reported symptoms as straining, splinting, constipation (defined by the Rome III criteria) [8], and/or dyschezia.

All patients underwent, before surgery, ultrasound and bimanual pelvic examination. In cases of suspected early endometrial or cervical cancer (exclusion criteria for the present study), staging magnetic resonance imaging or computed tomography was performed.

All surgeries were performed by the same surgeon (A.M.), an expert in urogynecology and laparoscopic pelvic surgery, assisted by a gynecological fellow.

For each patient, diagnostic, clinical, and surgical data were recorded. Anatomic surgical failure was defined by a POP-Q stage ≥ II at any site [7]. Operative time (OT) was defined as the interval between the incision start and closure. Bowel, bladder, ureteral, or vascular injuries were analyzed for intra-operative complications. Post-operative complications were studied, according to Clavien–Dindo’s (CD Grade) classification [9], during the first 30 days after surgery.

All patients completed, as part of our surgical protocol, the 2-year *Patient Global Impression of Improvement* (PGI-I) [10] and filled out, preoperatively and at 24-month follow-up, the *Pelvic Organ Prolapse Distress Inventory-6* (*POPDI-6*) [11], the *Pelvic Floor Impact Questionnaire-7* (PFIQ-7) [12], the *Female Sexual Distress Scale* (FSDS) [13], and the *Pelvic Organ Prolapse/Urinary Incontinence Sexual Questionnaire* (PISQ-12) [14].

In addition, all patients completed the *EuroQol* (EQ-5D) [15] to assess their general health-related quality of life. It comprises 5 domains (mobility, self-care, usual activities, pain/discomfort, and anxiety/depression), each one with 3 possible levels: no problems = level 1, some problems = level 2, and severe problems = level 3. A utility score (EQ-5D index) ranging from −0.38 (worst health state) to 1 (best health state) can be generated according to the preferences of the general Italian population. The EQ-5D includes a visual analog scale (VAS), ranging from 0 (the worst possible) to 100 (the best possible health status).

All of the administered questionnaires evaluate different aspects of a patient’s *QoL* linked to prolapse, urinary incontinence, sexual life, and general health-related quality of life.

The clinical management of the patients, as this was an observational and retrospective analysis, was not modified by this study. Our paper was, then, considered exempt from Institutional Review Board (IRB) approval from the local Ethics Committee. The present study was conducted in accordance with the Declaration of Helsinki. As part of our institutional protocol, written informed consent for the surgical procedure and the analysis of related data was obtained from all patients before the procedure.

### 2.4. Statistical Analysis

Our study is the first ever comparing the impact of *LSC plus LPP* on the quality of life, sexual function, and patient satisfaction rate in patients with POP and severe posterior compartment prolapse. This makes it impossible to use previous studies for the calculation of sample size. Nonparametric Mann–Whitney U and Wilcoxon tests were used, once we had tested the non-normal distribution of the clinical variables with the Kolmogorov–Smirnov test. Differences between data expressed as percentages were analyzed with Fisher’s exact test.

Results are expressed as median and percentile range (25th–75th) for continuous and as the number and percentage for categorical variables.

For statistical analyses, we used the Statistical Package for the Social Sciences, version 17.0 (SPSS Inc., Chicago, IL, USA). Statistical significance was set at *p* (two-sided) < 0.05.

## 3. Results

One hundred thirty-nine consecutive POP patients were retrospectively assessed for inclusion in our study. No missing data were reported for the studied patients. The STROBE diagram is shown in Figure 1.

No significant differences were observed between the groups in terms of the baseline characteristics of our populations, as shown in Table 1.

At the 2-year follow-up, as shown in Table 2, significant results were observed in terms of the successful outcome rate of the PGI-I (Group A versus B: 85.3% versus 67.1%; *p*: <0.05).

The POPDI-6 and PFIQ-7 resulted, in both studied populations, significantly reduced scores compared to the baseline. Anyway, no differences were found when analyzing the baseline and follow-up data between the groups.

Interestingly, at the 2-year evaluation, the FSDS and PISQ-12 resulted in a statistically significant reduction in Group A as compared to Group B (*p* <0.05) (Table 2).

**Figure 4:**
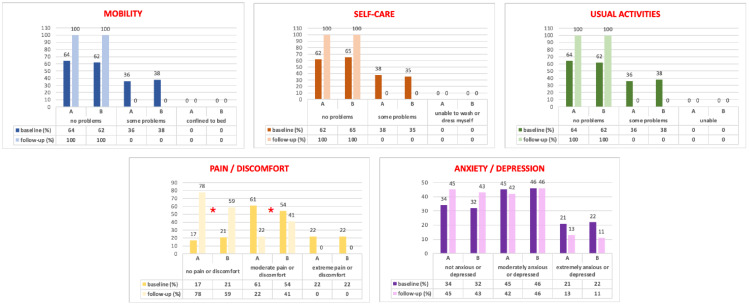
EQ-5D domains results at baseline and at the 2-year follow-up. *: *p* < 0.05 between the A and B Groups.

Figure 4 shows the EQ-5D domain distribution at baseline and at the 2-year follow-up. All EQ-5D indexes) and VAS scores (Table 2) significantly improved from baseline to the 2-year follow-up, but only in the pain/discomfort domains did we observe statistical differences between the groups, with a significant improvement in the LSC plus LPP patients (*p* < 0.05). However, in the anxiety/depression section, this finding did not reach statistical significance (Figure 4).

The studied perioperative and anatomical data are presented in Table 3. OT, intra-, and post-operative complications were found not to be statistically different between the populations.

We observed a single patient in the LSC-alone arm who developed a urinary tract infection at 2 weeks from surgery, which was successfully treated with antibiotics (CD Grade 1), and only one patient per group presented with fever on day two after surgery, resolved with paracetamol 1000 mg. We found no significant differences in terms of de novo dyspareunia and defecatory dysfunction between the groups. All symptoms had, anyway, spontaneous resolution within two weeks from hospitalization (Table 3).

We performed a urinary stress incontinence procedure, four months after surgery, in six patients in Group A (8%) and six in Group B (9%) (*p* > 0.05).

At the 2-year follow-up, as shown in Table 3, no vaginal mesh erosions were observed in the studied women.

Separately analyzing the anterior, apical, and posterior anatomical results, we have not found any differences in terms of POP-Q stage > II at any site [7], and the data of overall anatomical failures showed no statistical differences between the two populations. In particular, we observed one patient in both groups with an anterior vaginal recurrence and two patients in the LSC alone population with a posterior recurrence (Table 3).

Focusing on the posterior vaginal POP-Q points, the Ap point in Group A (median −2.3 versus −0.3 cm; *p* < 0.05) was found to be significantly improved. Also, genital hiatus (GH) measurements were significantly reduced in Group A patients when compared to standard LSC (median 1.7 versus 3.1 cm; *p* < 0.05) (Table 3).

The other POP-Q parameters did not show statistical differences between the populations.

## 4. Discussion

This is the first study in the literature reporting the impact of *LPP* combined with standard *LSC* in patients with severe posterior colpocele in terms of quality of life, sexual function, and patient satisfaction rate, with a 2-year follow-up.

Laparoscopic sacral colpopexy (*LSC*) is the gold standard approach for anterior/apical pelvic organ prolapse (POP), and when compared with vaginal procedures for apical suspension, it was linked with a significative reduction in anatomic recurrence or a need to repeat surgery at 2 years [1]. Regarding posterior prolapse, however, data from the literature show that, in terms of recurrence risk, vaginal fascial native tissue repair seems to be preferable to the laparoscopic prosthetic approach [2].

Posterior vaginal plication represents, therefore, the most appropriate technique for the treatment of an isolated posterior prolapse. It is, anyway, still unclear which surgical technique could be most appropriate for patients with a simultaneous presence of anterior–apical prolapse, for which the literature recommends an abdominal approach, or a severe posterior prolapse, for which guidelines recommend fascial vaginal surgery.

In the last decades, the FDA, starting from the literature data, have unquestionably stated that opening the vagina during prosthetic prolapse reconstruction surgery should be avoided and, in particular during sacral colpopexy, the risk of erosion statistically increases in cases of vaginal incision-opening for total hysterectomy [16].

In light of these observations, in a recent paper [3] we validated the *LPP plus LSC* technique as a new surgical approach to patients with anterior/apical prolapse associated with a concomitant severe posterior colpocele, demonstrating, with a 1-year follow-up, significant results in terms of anatomical repair, especially on the posterior compartment. In patients with anterior–apical and severe posterior pelvic prolapse, our surgical approach was found to match, in a single and concomitant laparoscopic approach, the anatomical and functional benefits of prosthetic surgery and of native tissue repair, avoiding the incision-opening of the vagina.

Regarding the primary endpoints of the present study, our data highlighted, with a 2-year follow-up, a significant improvement in quality of life, patient satisfaction rate, and sexual questionnaires in both studied populations from baseline, even if we observed a statistical difference in favor of *LPP plus LSC* versus *LSC alone* only in the sexual questionnaires (FSDS and PISQ-12) and the PGI-I *successful outcome* rates (Table 2).

Moreover, this is the first paper in the literature investigating, in a structured manner, women’s general health-related quality of life after *LPP plus LSC* with the EQ-5D. Both groups showed a statistical improvement in the EQ-5D distribution responses from baseline to follow-up. Only in the “*pain/discomfort*” domains did we statistically observe data in favor of Group A (Figure 4), probably linked to our anatomical results.

Anatomically, in fact, we confirmed, at 2 years, the data observed at the 12-month follow-up in our previous study [3]. We found in the *LSC plus LPP* Group a significant improvement in the Ap and GH POP-Q measurements in the absence of significant differences in terms of surgical failure between the studied populations [7]. These data were observed without statistical differences in terms of intra- and post-operative complications, vaginal erosion, or de novo dyspareunia/defecatory dysfunction rates (Table 3).

The present study can be considered in line with the new surgical research trends that are increasingly paying attention to quality of life and patient satisfaction rates [1,17]. Women who undergo pelvic floor dysfunction surgical procedures have a variety of desired subjective outcomes related to social roles, sexuality, and self-image. A successful POP surgery, therefore, can no longer only be based on anatomical repair but also on amelioration of the patient’s impaired quality of life, which is not always reflected in vaginal anatomical restoration [1,6].

At the 24-month follow-up, we observed with PGI-I a high degree of overall satisfaction (A versus B: *p* < 0.05). In fact, 85.3% (64/75) of *LPP* women showed a PGI-I *successful outcome* rate (“very much” and “much better” responses), data further increasing up to 93.3% when we included patients with a score of 3 (70/75). Interestingly, in the present study, we observed, at 2 years, data not so dissimilar from those observed in our previous paper at the 1-year follow-up (88.7% PGI-I *successful outcome* rate) [3]. We could explain these results by the significative positive effect of *LPP plus LSC* on posterior vaginal prolapse.

Furthermore, we also observed this persistent improvement in the patient satisfaction rate in our four cases of anatomical failure (Table 3), probably due to the mild degree of recurrent prolapse. Precisely, 100% (4/4) of these cases, in fact, were characterized by a recurrent stage II prolapse, which was asymptomatic for the patients.

Historically, transvaginal, transanal, transperineal, and abdominal approaches have been proposed for posterior compartment prolapse [18], even if vaginal native tissue repair was linked to a lower recurrence risk [2].

Anyway, while LSC is considered the gold standard approach for anterior and apical anatomy, this surgery has an important role in improving the GH size [19] and several studies have affirmed that it can restore the posterior compartment [3,18,19,20].

Our *LPP* technique, without vaginal incision, avoids the risk of mesh erosion and vaginal scars and, restoring the posterior vaginal anatomy, increases the benefits of sacral colpopexy on the patient’s sexual life [21], as shown by our FSDS and PISQ-12 data (Table 2).

The significant improvement in the Ap and GH POP-Q points at the 2-year follow-up, furthermore, could be considered an important outcome of our *posterior plication* approach. Correcting GH to <4 cm during prolapse repair, in fact, is linked to a significant reduction in long-term failure, as reported by Hill et al. in 2019 [22]. A prospective multicenter study will be necessary to evaluate whether the benefits of our LPP procedure can also be observed in patients with isolated posterior vaginal prolapse not requiring LSC.

Our FSDS, PISQ-12, and PGI-I data at the 2-year follow-up, in addition, strongly highlight the statistical improvement in the sexual quality of life after LPP plus LSC in women with severe posterior POP.

There are strengths and limitations of our study. Primarily, its single-center and retrospective design lead to the necessity of a multicenter trial to better verify our data. In particular, even if, as shown in Table 1, our populations were statistically homogeneous in terms of baseline characteristics, the non-randomized study of our populations represents a limitation of our analysis.

The systematic study of all women with validated questionnaires could be considered a strength of our paper. For the first time, moreover, general health-related quality of life was assessed with the EQ-5D for patients who underwent an *LPP plus LSC* approach.

The 24-month follow-up in the present paper also allowed us to better verify, at long-term, the data of our previous study [3].

Furthermore, even if it limits the generalizability of our data, the same expert operator performed all of the studied procedures, avoiding any bias related to inter-surgeon learning curves.

In addition, since *LSC* is a procedure with a high learning curve, and the additional laparoscopic stapling approach for *LPP* could increase the surgical difficulty of the procedure, a study expressly analyzing the LPP learning curve in a resident population could be of particular interest.

## 5. Conclusions

This is the first paper analyzing, with validated questionnaires, the quality of life, patient satisfaction rate, and sexual function among patients who underwent *LSC plus LPP* for severe posterior vaginal prolapse, with a follow-up time of 2 years.

This combined prosthetic and fascial laparoscopic technique can be considered a feasible approach during standard “two meshes” sacral colpopexy and is recommended in patients with apical/anterior prolapse and concomitant severe posterior colpocele. *Laparoscopic posterior vaginal plication*, in addition to improving the anatomical results of these patients, is associated, in fact, with a significant improvement in sexual and quality of life indexes.

## Figures and Tables

**Table 1:** Baseline characteristics of the studied populations.

	GROUP A (*LSC + LPP*) 75 pts	GROUP B (*LSC alone*) 64 pts	*p*
**Age (year), *median* (*range*)**	67 (62–74)	66 (60–72)	NS
**BMI, *median* (*range*)**	25 (22–26)	24 (21–26)	NS
**Nulliparous, *n* (%)**	5/75 (6)	4/64 (6)	NS
**Previous Abdominal Surgery, *n* (%)**	7/75 (9)	6/64 (9)	NS
**ASA score ≥ 2, *n* (%)**	8/75 (10)	8/64 (12)	NS
**POP-Q stage, *median* (*range*)**	3 (2–4)	3(2–4)	NS
**Aa, *median* (*range*)**	1.4 (1.1 to 2.4)	1.3 (1.1 to 2.3)	NS
**Ba, *median* (*range*)**	2.2 (2.1 to 3.7)	2.1 (2 to 3.8)	NS
**Ap, *median* (*range*)**	2.4 (1.7 to 3)	2.3 (1.5 to 2.8)	NS
**Bp, *median* (*range*)**	3.2 (1.1 to 3.9)	3.1 (1.1 to 3.7)	NS
**C, *median* (*range*)**	3.3 (2.7 to 4.6)	3.1 (2.6 to 4.5)	NS
**GH, *median* (*range*)**	4.6 (3.7 to 5.2)	4.7 (3.9 to 5.4)	NS

Legend: Two-sided significance level set at *p* < 0.05; NS: not significant; range: percentile range (25–75); BMI: body mass index; ASA score: American Society of Anesthesiologists score; POP-Q stages: Pelvic Organ Prolapse Quantification system scores Aa, Ba, Ap, Bp, C, and GH.

**Table 2:** Subjective outcomes.

	GROUP A (*LSC + LPP*) 75 pts	GROUP B (*LSC alone*) 64 pts	*p*
**PGI-I successful outcomes, *n* (%)**	64/75 (85.3)	43/64 (67.1)	<0.05
**POPDI-6**			
**Pre-Op, *median* (*range*)**	43 (35 to 54)	41 (32 to 49)	NS
**FU, *median* (*range*)**	7 (6 to 10) *	10 (7 to 11) *	NS
**PFIQ-7**			
**Pre-Op, *median* (*range*)**	14 (11 to 21)	15 (12 to 21)	NS
**FU, *median* (*range*)**	3 (2 to 4) *	4 (2 to 6) *	NS
**FSDS**			
**Pre-Op, *median* (*range*)**	38 (35 to 46)	39 (36 to 48)	NS
**FU, *median* (*range*)**	11 (7 to 13) *	21 (18 to 27) *	<0.05
**PISQ-12**			
**Pre-Op, *median* (*range*)**	42 (33 to 51)	38 (30 to 49)	NS
**FU, *median* (*range*)**	89 (78 to 96) *	62 (58 to 71) *	<0.05
**EQ-5D VAS**			
**Pre-Op, *median* (*range*)**	40 (30 to 60)	40 (30 to 50)	NS
**FU, *median* (*range*)**	80 (70 to 90) *	80 (70 to 90) *	NS

Legend: Two-sided significance level set at *p* < 0.05; NS: not significant; *: *p* < 0.05 between pre-op and follow-up; range: percentile range (25–75); PGI-I successful outcomes: “Patient Global Impression of Improvement” responses “very much” and “much better”; POPDI-6: “Pelvic Organ Prolapse Distress Inventory-6” (PFDI-20); PFIQ-7: “Pelvic Floor Impact Questionnaire-7”; FSDS: “Female Sexual Distress Scale”; PISQ-12: “Pelvic Organ Prolapse/Urinary Incontinence Sexual Questionnaire-12”; EQ-5D VAS: “EuroQol Visual Analog Scale”.

**Table 3:** Perioperative and anatomical outcomes.

	GROUP A (*LSC + LPP*) 75 pts	GROUP B (*LSC alone*) 64 pts	*p*
**Operative time (min), *median* (*range*)**	132 (115–141)	128 (111–131)	NS
**Intraoperative complications, *n* (%)**	0/0 (0)	0/0 (0)	NS
**Postoperative complications, *n* (%)**	1/75 (1)	2/64 (3)	NS
**De novo dyspareunia, *n* (%)**	3/75 (4)	3/64 (4)	NS
**De novo defecatory dysfunctions, *n* (%)**	1/75 (1)	1/64 (1)	NS
**Vaginal erosions, *n* (%)**	0/0 (0)	0/0 (0)	NS
**Anatomic failure (overall), *n* (%)**	1/75 (1)	3/64 (4)	NS
**Ap, *median* (*range*)**	−2.3 (−2.2 to −2.9)	−0.3 (−0.1 to −1.3)	<0.05
**Bp, *median* (*range*)**	−2.2 (−2 to −3)	−2.1 (−1.8 to −3)	NS
**GH, *median* (*range*)**	1.7 (1.2 to 2.4)	3.1 (2.8 to 4)	<0.05

Legend: Two-sided significance level set at *p* < 0.05; NS: not significant; range: percentile range (25–75); Ap, Bp, GH: Pelvic Organ Prolapse Quantification system scores.

## Data Availability

The data that support the findings of this study are available on request from the corresponding author.
